# Valorization of Olive Stone in Cement Mortars for Harmonized Applications

**DOI:** 10.3390/ma18225200

**Published:** 2025-11-17

**Authors:** Maria Martin-Morales, Gloria Maria Cuenca-Moyano, Maria Jose Martinez-Echevarria, Montserrat Zamorano, Monica Lopez-Alonso

**Affiliations:** 1 Department of Building Construction, ETS de Ingenieria de Edificacion, University of Granada, Campus de Fuentenueva s/n, 18071 Granada, Spain; mariam@ugr.es (M.M.-M.); gloriacuenca@ugr.es (G.M.C.-M.); 2 Department of Construction Engineering and Engineering Civil Projects, ETS Ingeniería de Caminos, Canales y Puertos, University of Granada, Campus de Fuentenueva s/n, 18071 Granada, Spain; mjmartinez@ugr.es; 3 Department of Civil Engineering, ETS Ingeniería de Caminos, Canales y Puertos, University of Granada, Campus de Fuentenueva s/n, 18071 Granada, Spain; zamorano@ugr.es

**Keywords:** cement mortars, olive stone, circular economy, waste valorization, standards

## Abstract

Nowadays construction sector continues to face major environmental challenges, largely due to the consumption of natural resources, energy, and water, as well as the generation of waste and emissions into the environment. In Andalusia (Spain), the olive oil industry plays a central role in the economy, generating large volumes of waste and by-products, including olive stones. Due to their physical characteristics, olive stones represent a potential substitute for conventional aggregates in cement mortars, which are not subjected to restrictive technical standards as concrete. This study evaluates the technical feasibility of cement mortars prepared by replacing 10% and 20% of conventional sand (by volume) with four different types of olive stones. Tests were carried out on setting times, consistency, density, and air content, in fresh state, as well as on capillarity, density, and flexural and compressive strengths, in hardened state, using a conventional mortar as reference, with favorable results. Mortars could be classified according to the harmonized standards for masonry, rendering and plastering, and flooring and screed mortars, and for the applications described in the Spanish Building Code (CTE). This progress in knowledge would further support the integration of the construction sector into the fields of sustainability and circular economy.

## 1. Introduction

The construction industry has a significant impact on both the economy and the environment [1]. Its economic contribution is considerable, accounting for approximately 5.5% of the European Union’s GDP, employing around 25 million people across more than 5 million companies [2]. At the same time, it is a major consumer of resources, using nearly 50% of raw materials and 36% of the total global final energy consumption [3,4]. Furthermore, it is responsible for a high share of harmful emissions into the atmosphere (23%), mainly CO_2_ [5], and for generating about 10 billion tons of waste annually [6].

Traditionally, the sector has prioritized productivity while largely neglecting the available strategies to reduce its environmental footprint [7]. In fact, it was not until the late 20th century that the first studies addressing the sustainability issues derived from construction activities began to emerge [8]. In this regard, the United Nations, through the Sustainable Development Goals (SDGs) established in 2015, emphasized the crucial role of the construction industry in achieving several of these objectives [7].

Within the framework of the circular economy strategy, the construction sector must adapt its production systems to incorporate resources derived from waste or by-products, using them as secondary raw materials. The aim is to minimize the consumption of natural resources, water, and energy, as well as to reduce waste generation and emissions into the environment [9,10,11,12,13].

The use of secondary raw materials in the production of cement mortars represents a promising opportunity for the sector. Mortars are among the most widely used materials in construction, and unlike concrete, they are not subject to the same high performance requirements or restrictive regulations. In this context, it is well known that sand is required in large quantities, accounting for 75–85% of the total weight of mortar in mixes with binder/sand ratios of 1:3 and 1:6, respectively. This has significant implications for the material’s environmental viability. Sand is the second most demanded natural resource worldwide, after water and ahead of fossil fuels, with approximately 85% of it destined for construction [14]. Consequently, its partial replacement with alternative granular materials has been identified as a strategic recommendation, helping improve the sector’s position in terms of sustainability.

The scientific literature reports numerous studies demonstrating the technical and environmental feasibility of partially or fully substituting natural aggregates in mortars with various by-products or secondary raw materials. The replacement of natural aggregates with inorganic recycled materials such as recycled aggregates [15,16], ceramic bricks [17,18], or glass [19,20] has been widely investigated, generally yielding positive results. However, studies on the use of unconventional organic aggregates are less common, although particularly relevant when aiming to enhance mortar properties such as lightness and thermal insulation. This potential has been observed in research involving granular biomass materials such as sawdust [21], cork [22], nutshells (walnut, pistachio, hazelnut, and peanut) [23], mussel shells [24], or palm kernel shells [25].

From this perspective, olive stones stand out as a potential substitute for conventional sand due to their physical properties, including low density, rough surface texture, and good mechanical resistance. Olive stones are a by-product of the olive oil industry, which has a strong presence in Mediterranean countries, particularly in Andalusia, Spain. Owing to their high calorific value, olive stones are widely used as biofuel in thermal and thermoelectric energy production in boilers and power plants. However, this application poses a severe environmental risk, as small-diameter particles may fail to combust fully, being released into the atmosphere as suspended particulate matter [26].

According to data from the Spanish Ministry of Agriculture and the Environment [27], Spain is the world leader in olive production, with 2.75 million hectares under cultivation. Of this production, 93% is destined for olive oil and 7% for table olives, amounting to a total of 8138 million tons of olives. Considering that the stone represents approximately 20% of the total fruit weight [28], olive stone production in Spain is estimated at around 628 million tons per year.

The scientific literature on the use of olive stones as sand replacement in mortars remains scarce. In 2013, Barreca and Fichera [29] pioneered the use of olive stones in lime–cement mortars, achieving reductions in thermal conductivity of 76% and in density of 30% with a 70% replacement (by weight), compared to conventional mortars. In 2017, Del Río Merino [30] experimented with entire, crushed, and calcined olive stones in lightweight cement mortars as substitutes for traditional expanded clay, although crushed olive stones were eventually discarded due to low mechanical strength. Cheboub et al. (2020) [31] later studied lightweight self-compacting mortars incorporating up to 100% olive kernel shells as sand replacement, reporting significant improvements on physic properties despite substantial reductions in mechanical strength. Specifically, they achieved a weight reduction in the mortars by more than 40%, reaching a density of 1410 kg/m^3^ and a thermal conductivity of 0.326 W/m·K, which represents an improvement of 77%. More recently, Los Santos Ortega and colleagues investigated the potential of ground olive stones in construction, applying mortars in the production of sustainable façade bricks to improve thermal insulation. Substitution levels of up to 30% by volume achieved energy demand savings (heating and cooling) of 0.938 kWh/m^2^·year compared to reference bricks, equivalent to a 2.23% reduction per square meter of façade [32]. Their studies also indicated significant economic returns when used olive stone in cement mortar bricks as a partial replacement of natural sand between 5% and 15%, due to reduced conventional energy consumption for heating and cooling, as well as decreased environmental impacts in the medium (10–20 years) [33]. In the long term, for a simulation period of 35 years [34], the LCA conducted on mortars with 20% olive stone-doped cement reported a reduction in CO_2_ emissions of 137.9%, preventing the release of 319.43 kg CO_2_ eq/m^3^ of mortar into the atmosphere and avoiding the consumption of 3221.1 MJ/m^3^ of fossil fuels. In 2022, Ferreiro-Cabello [35] investigated cement mortars produced with nine different dosages of ground olive stones and three types of cement. Tests on consistency, density, and mechanical strength indicated that a maximum of 30% olive stone (by volume) could be incorporated without compromising mortar performance. Although lightweight mortars were not achieved, the dry density was reduced by 85%, while the compressive strength dropped drastically by up to 70%. Finally, Boubakour et al. (2023) [36] also reported that substitutions above 30% compromised consistency, density, and mechanical properties. In this case, using olive stone replacement ratios between 5% and 30%, it was found that the mortar consistency remained within the plastic range, while the density decreased significantly from a 20% replacement onwards, although without reaching the lightweight mortar category. However, the flexural and compressive strengths were severely affected even at the lowest replacement levels, showing reductions of approximately 40% to 70%. Nevertheless, the mortars displayed improved durability and long-term performance, particularly a marked reduction in chloride penetration depth across all ages, ranged between 44% and 55%.

Although reductions in mechanical strength have been consistently observed, all previous studies confirm that olive stones decrease mortar density and thermal conductivity [29,31,32], providing added value for potential industrial applications. However, there is still a lack of in-depth research addressing relevant regulatory aspects for the practical use of such mortars—such as workability and durability—as well as the influence of olive stone particle size.

Therefore, the aim of this study is to advance the technical evaluation of olive stone valorization as a partial substitute for conventional sand in cement mortars, assessing compliance with regulatory requirements specified in the harmonized European standards for masonry mortars (UNE-EN 998-1 [37], rendering and plastering mortars (UNE-EN 998-1) [38], and flooring and screed mortars (UNE-EN 13813) [39] and the Spanish Technical Building Code (CTE) [40]. This contribution would yield dual sustainability benefits: for the construction sector, by reducing reliance on natural aggregates, and for the olive oil sector, by mitigating waste generation and airborne particulate emissions.

## 2. Materials and Methods

### 2.1. Materials

**Figure 1:**
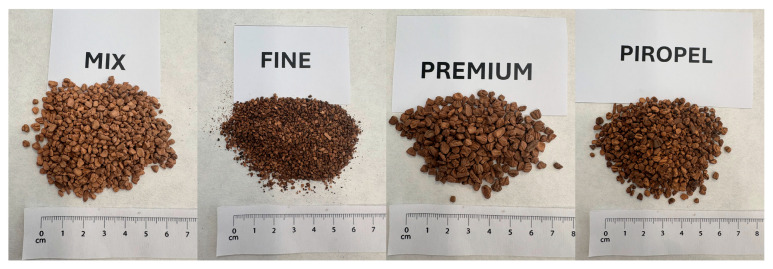
Olive stones.

**Figure 2:**
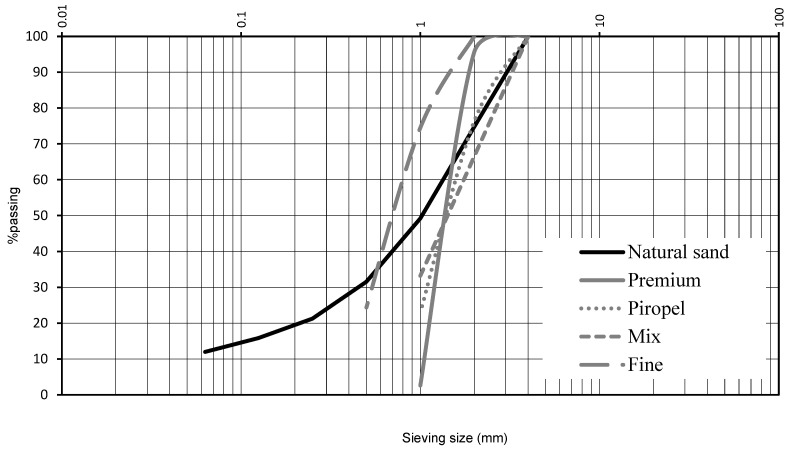
Particle size distribution of the aggregates.

The masonry mortars studied were prepared with the following materials:

- Natural sand: Conventional limestone–dolomitic sand sourced from an aggregate plant in the south of Spain, province of Granada, meeting the requirements of UNE EN 13139 for aggregates for mortars [41]. The sand had a particle size of 0/4 mm and a density of 2.77 g/cm^3^. Its particle size distribution is shown in Figure 1;
- Filler: Limestone–dolomitic filler from an aggregate plant in Granada, also complying with UNE EN 13139 [41], with a density of 2.71 g/cm^3^;
- Cement: Portland cement CEM I 42.5 R supplied by Cementos Portland Valderrivas Group (Madrid, Spain), conforming to the requirements of the Spanish cement reception regulation RC-16 [42], with a density of 3.01 g/cm^3^;
- Admixture: High-performance superplasticizer and water-reducer MASTEREASE 3530 (Master Builders Solutions, Madrid, Spain), with a density of 1.06 g/cm^3^;
- Water: Potable water;

Olive stones: Four different types of olive stone supplied by Peláez Renovables S.L., located in Bailén, Jaén (Spain) (Figure 1). The stones were obtained through a process of drying, cleaning, and particle size separation of residues from olive oil extraction in mills, aimed at producing materials with different energy yields. The physical characteristics of the commercial products Mix, Fine, Premium, and Piropel are summarized in Table 1, and their particle size distributions are shown in Figure 2. As can be observed, although all four types of olive stones are classified as fine aggregates, they exhibit considerably different particle sizes, particle size distributions, and water absorption capacities.

### 2.2. Sample Designation

One reference mortar (C) and eigth mortars incorporating olive stone as aggregate replacement were produced. The mortars were designated following the pattern X–Y, where X indicates the type of olive stone (M = Mix, Pr = Premium, F = Fine, and Pi = Piropel) and Y indicates the percentage of natural aggregate replaced by olive stone (10% or 20% by volume).

### 2.3. Mortar Mix Proportions

The mortars were prepared using a conventional dosage: cement-to-sand ratio of 1:8, water-to-cement ratio of 1.2, filler content of 13% by weight of natural aggregate, and 1% of admixture.

Table 2 summarizes the quantities of components per batch, considering that olive stone replaced natural aggregate by volume. Due to the high water absorption capacity of olive stone (see Table 1), and to avoid consistency variations, the olive stone was incorporated into the solid mix after being pre-saturated to 80% of its 24 h absorption capacity 10 min before mixing with the other components. This pre-wetting water also favored subsequent self-curing of the mortar. The pre-wetting water was subtracted from the amount of water added for kneading, so the effective amount of water remains constant.

### 2.4. Mortar Testing

The mortars were prepared using a conventional dosage: cement-to-sand ratio of 1:8, water-to-cement ratio of 1.2, filler content of 13% by weight of natural aggregate, and 1% of admixture.

The mortars were manufactured in accordance with UNE-EN 998-2 [38], and the following properties, shown in Table 3 with the corresponding standards, were assessed:

- In fresh state: setting times, consistency, bulk density, and entrained air content;
- In hardened state: capillary water absorption, bulk density, and flexural and compressive strengths.

To evaluate the potential effect of olive stone on cement setting, setting time tests were performed according to UNE-EN 196-3 [43], using not only distilled water but also eluates obtained by leaching olive stones. Specifically, two types of eluates were prepared with Fine (F) and Premium (Pr) olive stones, which together cover the full sand particle size range. A liquid-to-solid ratio of 2 L/kg was applied. The eluates were prepared in deionized water (DW) and in deionized water saturated with lime (DCW, 7.34 g Ca(OH)_2_ per 2 L of water), in order to simulate extreme conditions. The eluates were filtered after 24 h and subsequently used in the test.

## 3. Results and Discussions

**Figure 3:**
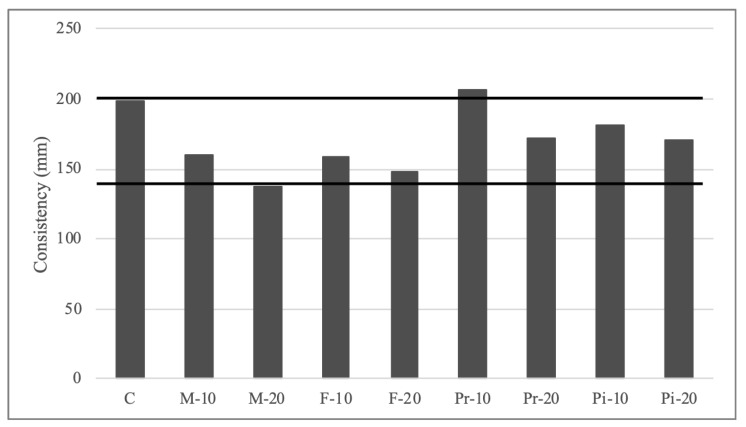
Results of consistency on mortars.

**Figure 4:**
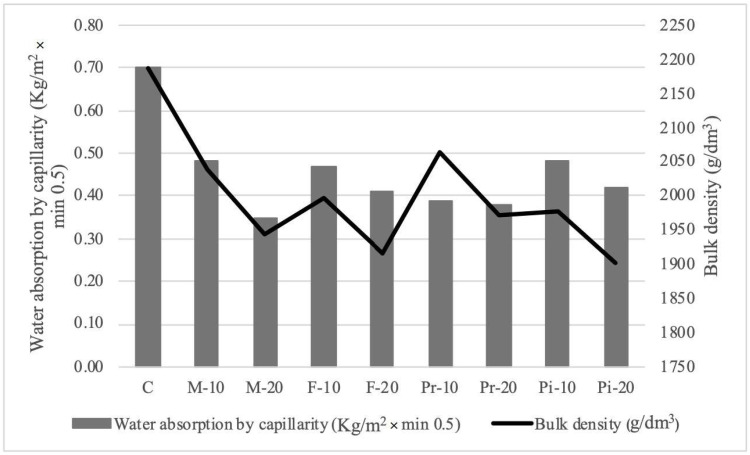
Results of water absorption by capillarity and dry bulk density on mortars.

**Figure 5:**
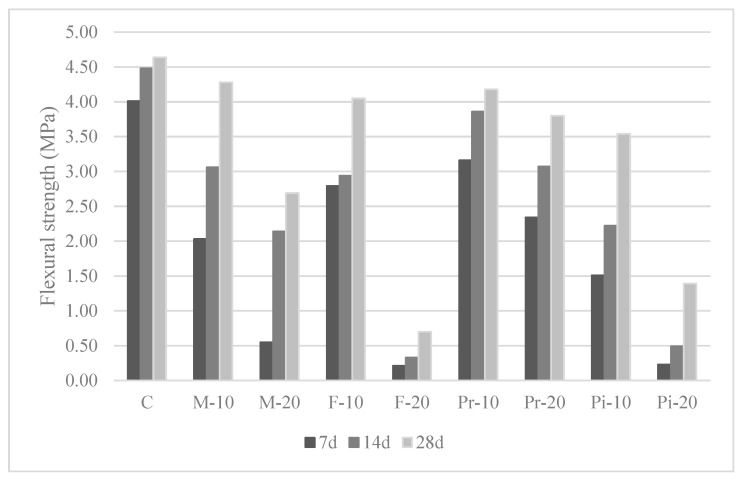
Results of flexural strength on mortars.

**Figure 6:**
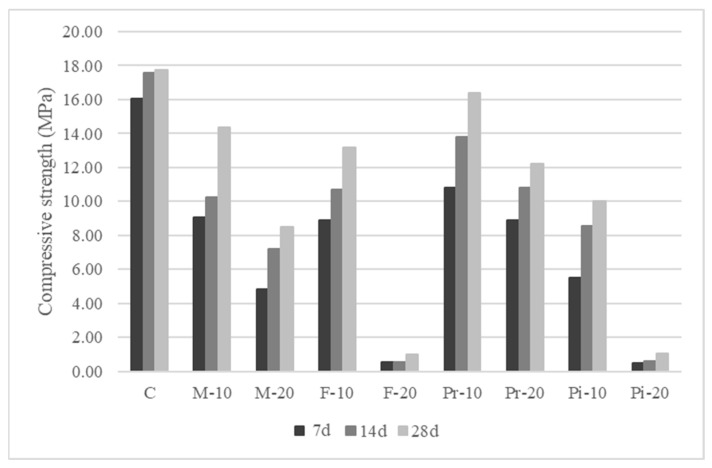
Results of compressive strength on mortars.

The experimental results of setting times (Table 4), consistency (Figure 3), density and entrained air content (Table 5) of mortars prepared in the fresh state, as well as water absorption by capillarity (Figure 4), apparent density (Figure 4), and mechanical strengths in flexion (Figure 5) and compression (Figure 6) in the cured state, are presented and discussed below. In the following subsections, the test results are discussed, considering for each of them the arithmetic mean of 3 samples.

### 3.1. Results of Settings Times

Table 4 summarizes the results of the setting time test carried out on normal consistency pastes [43] prepared for each type of eluate, as well as on a normal consistency paste prepared with deionized water as a reference, which define the initial and final setting times as well as the duration of the plastic state.

Contrary to expectations, all the setting scenarios show shorter initial setting times than the reference (195 min), reduced by between 15% and 45%, with the eluate saturated in calcium hydroxide (DCW) producing the greatest setting accelerations for both bone types. On the other hand, the duration of the plastic state is extended by between 10 and 60 min compared to the reference (105 min). However, each bone type behaved differently. For Premium bone, practically no difference is observed in the duration of the plastic state depending on the eluate type, while for Fine bone, when prepared with distilled water, the plastic state increases to 190 min, and when prepared with lime-saturated water, both the initial and final setting times are drastically shortened, resulting in a plastic state very similar to that of the reference mortar, extended by only 10 min. Therefore, it can be concluded that mortar made with olive stone contributes to improving the workability for the operator since it can be applied earlier than conventional mortar, with an extended applicability time.

### 3.2. Consistency

This property can be considered a priority in this type of material since, beyond other physical aspects, mortars need to be workable to facilitate their application as well as to promote physical adhesion with porous substrates.

Figure 3 shows the consistency results of the studied mortars measured using a flow table [44]. As can be observed, except for mortar M-20 (138 mm) and mortar Pr-10 (206.25 mm), all the mortars studied fall within the range of plastic consistency (140–200 mm) [50], meaning these mortars are perfectly suitable for use as masonry mortars. The rough nature of vegetal particles and their considerable water absorption capacity make it difficult to obtain workable pastes, hence the pre-soaking technique described earlier was proposed to enhance plasticity without compromising cement setting. Even so, replacements above 20% could not be achieved.

Except for mortar Pr-10, which shows a slight improvement in consistency of 3.5% compared to the reference mortar (199.25 mm), the incorporation of vegetal granular material reduces workability, ranging from 8.8% for mortar Pi-10 to 30.7% for mortar M-20. From the perspective of bone type, it can be seen that water absorption capacity is directly related to mortar consistency. Mix (20.22%) and Fine (26.79%) stones most compromise this property, while Piropel (21.23%) and Premium (19.74%) stones show consistencies closest to the reference mortar.

Similar reductions in consistency were found in the work of Boubakour [36]. These authors, starting from a fluid consistency reference mortar (maintained up to 10% substitution, without pre-soaking), could not achieve workable pastes with more than 30% substitution.

### 3.3. Fresh State Apparent Density

The apparent density of mortars in the fresh state is related to the component materials and entrained air content, such that lighter mortars are more workable [50]. As expected, Table 5 shows a proportional decrease in apparent density as the amount of vegetal granular material increases. This decrease ranges from 3.2% for mortar M-10 to 8.4% for mortar F-20, compared to the reference mortar (2294 g/dm^3^). Moreover, Mix, Premium, and Piropel stones yield very similar densities for both substitution levels, due to their maximum particle size of 4 mm and similar gradation.

### 3.4. Entrained Air Content

Table 5 shows the entrained air results, which increase proportionally with substitution percentage. No significant differences appear when Mix, Premium, and Piropel stones are used, with variations ranging from a 5.7% decrease for mortar Pi-10 to a 4.6% increase for mortar M-20, compared to the reference (4.35%). However, Fine bone considerably increases this parameter by 23.0% and 37.9% for 10% and 20% substitution, respectively.

Nevertheless, the entrained air contents found are generally low. The Spanish cement standard RC-16 [42] recommends values between 8% and 22% for fresh masonry cement mortars. These results could be attributed to factors such as the plasticizer additive, the good packing achieved by continuous granulometry, the high fines content (<0.063 mm), and adequate compaction. Entrained air will also increase porosity in the hardened mortar. Cheboub et al. (2020) [31], via SEM analysis, observed higher water absorption capacities in substituted mortars due to this effect.

### 3.5. Water Absorption by Capillarity

This parameter indirectly measures mortar durability in the hardened state, since water uptake by surface contact may cause undesirable particle/salt transport affecting durability. Thus, higher compactness reduces capillary absorption [50].

As seen in Figure 4, and contrary to expectations, substituted mortars show lower water absorption than the reference (0.70 kg/m^2^·min^0.5^), decreasing proportionally with substitution (31.4% for M-10 and Pi-10 mortars, and 50% for M-20). The Mix bone mortars exhibit the most extreme absorption values, while Premium bone mortars show practically no difference compared to the reference.

These results may be due to pore structures that, due to larger size and/or lower connectivity, prevent water capillary rise, making them suitable for humid climates. According to the Spanish Building Code (CTE) [40] in the Basic Document on Health (DB HS1), only mortars M-20, Pr-10, and Pr-20 can be classified as medium water-resistance masonry mortars (c ≤ 0.4 kg/m^2^·min^0.5^). Furthermore, the reduced water absorption would not compromise thermal insulation [29,31,33], nor durability against freeze–thaw and salt crystallization cycles.

While some authors [36] have found similar results (attributed to greater compactness), others [31] reported higher absorption with substitution, attributed to the hydrophilic nature of vegetal particles and porosity in the ITZ between olive stone and cement paste. It is important to emphasize that none of the studies applied the pre-wetting technique to minimize the absorption of mixing water by the vegetal material, which would otherwise lead to the formation of a more porous interfacial transition zone (ITZ). Thus, these results are inconclusive, requiring further study of the pore structure in such mortars.

### 3.6. Hardened State Apparent Density

Figure 4 also shows the bulk density of hardened mortars, which clearly decreases proportionally as sand is replaced by olive stone, due to their lower density and the air voids created in the matrix [31]. Compared to the reference mortar (2188 g/dm^3^), the decrease is modest: from 5.6% (Pr-10) to 13.1% (Pi-20). The stones reducing density most are, in order, Fine and Piropel, followed by Mix and Premium. This reduction aligns with particle size distributions: Fine and Piropel stones have finer particles, while Mix and Premium have coarser ones.

Despite the reduced densities, none of the mortars can be classified as lightweight since they remain above 1300 g/dm^3^, the UNE-EN 998-2 [38] threshold. These results agree with other studies [31,33,35], which report only moderate weight reduction. Achieving true lightweight mortar requires higher substitution levels: Ferreiro-Cabello [35] achieved 1226 g/dm^3^ at 50% substitution, though with reduced stability and workability.

Although it was not possible to produce lightweight mortars, these results should be understood positively since, as has been confirmed, the mortars studied can contribute to the thermal insulation of buildings [29,31,33], while also reducing the load applied to the structure.

### 3.7. Flexural Strength

The mechanical strength of the mortars, determined on prismatic specimens of 4 × 4 × 16 cm first tested under flexure and subsequently with both halves subjected to compression, showed the expected gradual and proportional reduction as the substitution percentages increased (Figure 5 and Figure 6).

As observed in Figure 5, which presents flexural strength at 7, 14, and 28 days, there was no significant loss of strength at 28 days compared to the reference mortar (4.64 MPa) for all 10% substitutions, with values ranging between 23.7% (Pi-10) and 7.8% (M-10). However, strength decreased considerably at 20% substitution, with reductions from 18.1% (Pr-20) to a dramatic 84.1% (F-20). The delay in setting caused by the incorporation of vegetal granular material was also confirmed, manifested in the low strength development at early ages. While the reference mortar reached 86.4% of its final strength at 7 days and 96.6% at 14 days, the substituted mortars did not exceed 69% at 7 days or 93% at 14 days. In general, these delays were more pronounced at 20% substitution due to the greater presence of vegetal particles.

These decreases in flexural strength have also been reported by other authors [31,35], attributed to the poor adhesion of these aggregates to the cement matrix, as observed in SEM analysis [31]. Considering the influence of aggregate type, the 10% substitutions showed moderate reductions for Mix mortars (7.8%), followed by Premium (9.9%) and Fine (12.7%), with higher losses for Piropel (23.7%). At 20% substitution, reductions became much more significant, ranging from 18.1% (Pr-20) to 84.9% (F-20). Overall, the best performance was achieved with Premium aggregates, followed by Mix, while Piropel and Fine aggregates proved unsuitable for 20% replacement.

### 3.8. Compressive Strength

Figure 6 presents compressive strength at 7, 14, and 28 days. At 28 days, a wider range of strength loss was observed relative to the reference mortar (17.75 MPa), varying between 7.7% (Pr-10) and 93.9% (Pi-20), with significantly higher losses at 20% substitution. Mortars with Fine and Piropel aggregates proved practically unfeasible due to their very low strength. These results again confirm the setting delay caused by the replacement of conventional sand with olive stone aggregates, especially at 20% substitution. Although the relative strength development compared to the reference mortar followed similar trends as in flexural tests (45–72.6% at 7 days, and 53.7–88.2% at 14 days for Pi-20 and Pr-20, respectively), the final 28-day losses were more severe. Except for the 10% Premium mortar, which showed a relatively low compressive strength loss (7.7%), reductions were substantial, ranging from 19.1% (M-10) to 94.5% (F-20).

These reductions were similar to those reported by other authors [31,33,36], attributed to the physical and geometrical properties of olive stone particles, which create local weaknesses in the cement matrix [31], thus confirming previous findings that mortars with more than 30% replacement are technically unfeasible [35,36]. As with flexural tests, aggregate type strongly influenced performance, with Premium and Mix mortars performing best for both substitution levels, followed by F-10 and Pi-10, while 20% substitutions of these latter proved completely unviable. These outcomes can be explained by the particle size distribution of the different olive stones, with Premium and Mix (4 mm maximum size and no fines) producing the highest strength.

From a regulatory standpoint, except for mortars produced with 20% Fine bones, all mortars studied can be classified according to their compressive strength under the harmonized standards UNE-EN 998-1 [37], UNE-EN 998-2 [38], and UNE-EN 13813 [39], and for the applications described in the Spanish Building Technical Code (CTE) [40].

### 3.9. Mortar Classification and Applications

Cement mortars can be classified according to their properties as masonry mortars for general use, thin-joint mortars, or mortars for bonding masonry units in walls, pillars, and partitions, as described in UNE-EN 998-2 [38]. According to this standard, and based on 28-day compressive strength, the mortars studied can be classified in the strength classes shown in Table 6, where M denotes the strength class (MPa).

In line with this classification, the Spanish Building Technical Code (CTE) [40], through its Basic Document on Structural Safety in Masonry (DB SE-F), specifies the requirements for façade elements to ensure structural stability. It allows the studied mortars to be used in ordinary walls (OW) when classified as at least M1, and in reinforced walls (RW), pre-stressed walls (PW) and thin-joint applications (TJ) when classified at least as M4. Furthermore, to ensure resistance to water penetration in joints of masonry units in external façade panels (EFP), the Basic Document on Health (DB HS1) requires mortars to be classified as M5 or M7.5, with capillary water absorption ≤ 0.40 kg/m^2^·min^0.5^. Table 6 summarizes these applications.

Likewise, the mortars studied can be used in the applications described in the harmonized standard UNE-EN 998-1 [37], which refers to rendering mortars (for external use) and plastering mortars (for internal use) applied to walls, ceilings, columns, and partitions. For this purpose, they must be classified according to their 28-day compressive strength into the categories CS I (0.4–2.5 N/mm^2^), CS II (1.5–5.0 N/mm^2^), CS III (3.5–7.5 N/mm^2^), or CS IV (>6 N/mm^2^), and according to capillary water absorption into categories W0 (unspecified), W1 (<0.4 kg/m^2^·min^0.5^), or W2 (<0.2 kg/m^2^·min^0.5^, using water-repellent admixture).

The Spanish Building Technical Code (CTE) [40], in its Basic Document on Health (DB HS1), specifies—based on these parameters—the requirements that mortars used as continuous exterior and intermediate renders of the façade external layer (MFR: mortar façade render) must meet to ensure adequate resistance to water penetration, depending on rainfall and wind exposure. Table 7 presents the classification of the mortars studied according to these requirements and their applications in line with such demands.

It is interesting to note that, despite the loss in mechanical performance of the mortars studied, they can still be employed in specific applications where mechanical properties are not of primary importance. In this regard, as established by the Spanish Building Technical Code (CTE) [40], within its Basic Document on Structural Safety in Masonry (DB SE-F), and with the exception of mortars F-20 and Pi-10, the mortars tested can be used for the manufacture of mortar blocks and bricks (MBB) for masonry, as they meet the minimum required strength of 5 MPa.

Finally, according to the harmonized standard UNE-EN 13813 [39], these mortars can also be used as flooring and screed cement mortars, classified according to their compressive strength. Table 8 summarizes these other uses.

## 4. Conclusions

Based on the tests carried out in this study, and despite the influence of the amount and type of olive stone used, it can be confirmed that its incorporation as a secondary raw material in the manufacture of cement mortars, partially replacing conventional aggregate, is technically feasible, thus contributing to construction sustainability and the circular economy.

The four types of olive stones used in the study show grain sizes and water absorption capacities that condition the behavior of mortars in both fresh and hardened states. The results obtained from the study have led to the following specific conclusions:

Olive stone produces significant modifications in cement setting times, with an advance in the initial setting of up to 45% compared to the reference mortar and an extension of the plastic state of up to 60 min. These alterations are more pronounced when Fine-type bones are used, as they are smaller in size and therefore have a larger specific surface area to react.

In the fresh state, the influence of both the content and type of olive stone was confirmed, with appreciable and expected modifications in consistency, density, and air content. However, it is noteworthy that the nature of the vegetal granular material strongly affects the consistency of fresh mortar, as pre-wetting is required before mixing with the other solid components to achieve the required plastic consistency. With substitution levels above 20%, mortars fall into the dry consistency range. Nevertheless, the entrained air content of the mortars, although below the range established in the Spanish cement instruction RC-16, was very similar to that of the reference mortar, except when Fine-type stones were used, which caused an increase of up to 37.9%.

In the hardened state, capillary water absorption, contrary to expectations, decreased considerably in the substituted mortars—up to 50% compared to the reference mortar—and proportionally with higher substitution levels, except for the Premium type, which showed no difference at either substitution level. This suggests a considerable improvement in the durability of mortars against phenomena associated with water flow within the material, such as freeze–thaw damage and salt crystallization. These results allowed the mortars made with Premium and Mix stones at 20% substitution to be classified as reduced-capillarity mortars (<0.4 kg/m^2^·min^0.5^), according to UNE-EN 998-1 and for the applications described in the Spanish Technical Building Code (CTE). Although further study of the pore structure of the hardened material is needed, this behavior could probably be attributed to the generation of a coarser and/or less interconnected capillary network.

Mortar density in the hardened state, as in the fresh state, followed the expected trend with small reductions of up to 13.1%, which did not allow mortars to be classified as lightweight (>1300 g/cm^3^). However, these reductions would significantly improve thermal insulation, as reported in the few existing studies referenced. The vegetal aggregates that most improved this behavior were Piropel and Fine stones, which have smaller grain sizes.

Finally, mechanical strengths were proportionally affected by the amount of vegetal granular material replaced, highlighting, on the one hand, the delay in strength development at early ages (7 and 14 days) for both compression and flexural strength, which corroborates the setting-time results. On the other hand, 10% substitutions produced moderate decreases in 28-day strengths across all mortars (up to 23.7% in flexural and 43.5% in compressive strength), while 20% substitutions produced greater reductions (up to 42.0% in flexural and 52.2% in compressive strength), resulting in Fine and Piropel mortars being invalid at this percentage. Consequently, based on 28-day compressive strength, the mortars studied could be classified as masonry mortars (UNE-EN 998-2), rendering and plastering mortars (UNE-EN 998-1), and flooring and screed mortars (UNE-EN 13813), for the applications described in the Spanish Technical Building Code (CTE).

In conclusion, the study results show that 10% substitution hardly compromises most of the tested parameters, while the olive stone providing the best mortar performance is the Premium type, followed by the Mix type, whereas Piropel and, lastly, Fine produced the worst results.

## Figures and Tables

**Table 1:** Physical characteristics of olive stones.

Olive Stone Type	Particle Size (mm)	Water Absorption at 24 h (%)	Density (g/cm^3^)
Mix	1–4	20.22	1.33
Fine	0–2	26.79	1.33
Premium	2–4	19.74	1.28
Piropel	0.5–4	21.23	1.34

**Table 2:** Mortar mix proportions per batch.

	Sand (g)	Filler (g)	Olive Stone (g)	Cement (g)	Water (g)	Admixture (g)
C	2657.50	345.50	0.00	328.00	393.60	3.28
M-10	2391.72	345.50	156.89	328.00	393.60	3.28
M-20	2125.97	345.50	314.10	328.00	393.60	3.28
F-10	2391.72	345.50	164.73	328.00	393.60	3.28
F-20	2125.97	345.50	329.79	328.00	393.60	3.28
Pr-10	2391.72	345.50	148.51	328.00	393.60	3.28
Pr-20	2125.97	345.50	297.32	328.00	393.60	3.28
Pi-10	2391.72	345.50	157.74	328.00	393.60	3.28
Pi-20	2125.97	345.50	315.49	328.00	393.60	3.28

**Table 3:** Tests performed on mortars and corresponding standards.

**Fresh State**	
Setting time	UNE-EN 196-3 [43]
Consistency	UNE-EN 1015-3 [44]
Bulk density in fresh state	UNE-EN 1015-6 [45]
Air content	UNE-EN 1015-7 [46]
**Hardened state**	
Capillary water absorption	UNE-EN 1015-18 [47]
Bulk density in hardened state	UNE-EN 1015-10 [48]
Mechanical strengths	UNE-EN 1015-11 [49]

**Table 4:** Setting times.

Paste	Initial Setting Time (min)	Final Setting Time (min)	Plastic State Duration (min)
Reference	195	300	105
DW + Pr	165	325	160
DCW + Pr	155	320	165
DW + F	140	330	190

**Table 5:** Results of tests carried out on mortars in the fresh state.

	Apparent Density in Fresh State (Kg/m^3^)	Entrained Air Content (%)
C	2294	4.35
M-10	2221	4.40
M-20	2130	4.55
F-10	2172	5.35
F-20	2101	6.00
Pr-10	2200	4.25
Pr-20	2133	4.35
Pi-10	2201	4.10
Pi-20	2102	4.40

**Table 6:** Classification of mortars according to UNE-EN 998-2 [38] and applications according to the Spanish Building Technical Code (CTE) [40].

	Compressive Strength at 28 Days (MPa)	Classification	Application According to CTE DB SE-F	Capillary Water Absorption (kg/m^2^·min^0.5^)	Application According to CTE DB HS1
C	17.75	M 15	OW, RW, PW, TJ	0.70	Without classification
M-10	14.36	M 10	OW, RW, PW, TJ	0.48	Without classification
M-20	8.49	M 7.5	OW, RW, PW, TJ	0.35	EFP
F-10	13.17	M 10	OW, RW, PW, TJ	0.47	Without classification
F-20	0.98	Without classification	Without classification	0.41	Without classification
Pr-10	16.39	M 15	OW, RW, PW, TJ	0.39	EFP
Pr-20	12.24	M 10	OW, RW, PW, TJ	0.38	EFP
Pi-10	10.03	M 10	OW, RW, PW, TJ	0.48	Without classification
Pi-20	1.08	M 1	OW	0.42	Without classification

**Table 7:** Classification of mortars according to UNE-EN 998-1 [37] and applications according to the Spanish Building Technical Code (CTE) [40].

	Compressive Strength at 28 Days (MPa)	Classification	Capillary Water Absorption (kg/m^2^·min^0.5^)	Application According to CTE DB HS1
C	17.75	CS IV	0.70	Without classification
M-10	14.36	CS IV	0.48	Without classification
M-20	8.49	CS IV	0.35	MFR
F-10	13.17	CS IV	0.47	Without classification
F-20	0.98	CS I	0.41	Without classification
Pr-10	16.39	CS IV	0.39	MFR
Pr-20	12.24	CS IV	0.38	MFR
Pi-10	10.03	CS IV	0.48	Without classification
Pi-20	1.08	CS I	0.42	Without classification

**Table 8:** Other uses and classification of mortars.

	Compressive Strength at 28 Days (MPa)	Application According to CTE DB F	Compressive Strength Classification of Flooring and Screed Cement Mortar (UNE EN 13813)
C	17.75	MBB	C16
M-10	14.36	MBB	C12
M-20	8.49	MBB	C7
F-10	13.17	MBB	C12
F-20	0.98	Without classification	Without classification
Pr-10	16.39	MBB	C16
Pr-20	12.24	MBB	C12
Pi-10	10.03	MBB	C7
Pi-20	1.08	Without classification	Without classification

## Data Availability

The original contributions presented in this study are included in the article. Further inquiries can be directed to the corresponding author.
